# A versatile 2A peptide-based bicistronic protein expressing platform for the industrial cellulase producing fungus, *Trichoderma reesei*

**DOI:** 10.1186/s13068-017-0710-7

**Published:** 2017-02-06

**Authors:** Venkataramanan Subramanian, Logan A. Schuster, Kyle T. Moore, Larry E. Taylor, John O. Baker, Todd A. Vander Wall, Jeffrey G. Linger, Michael E. Himmel, Stephen R. Decker

**Affiliations:** 1 Biosciences Center, National Renewable Energy Laboratory, 15013 Denver West Parkway, Golden, CO 80401 USA; 2 National Bioenergy Center, National Renewable Energy Laboratory, 15013 Denver West Parkway, Golden, CO 80401 USA

**Keywords:** *Trichoderma reesei*, Foot-and-mouth disease virus (FMDV) 2A peptide, Protein expression, Cellobiohydrolase, Fungus, Biomass hydrolysis, Green fluorescence protein

## Abstract

**Background:**

The industrial workhorse fungus, *Trichoderma reesei*, is typically exploited for its ability to produce cellulase enzymes, whereas use of this fungus for over-expression of other proteins (homologous and heterologous) is still very limited. Identifying transformants expressing target protein is a tedious task due to low transformation efficiency, combined with highly variable expression levels between transformants. Routine methods for identification include PCR-based analysis, western blotting, or crude activity screening, all of which are time-consuming techniques. To simplify this screening, we have adapted the 2A peptide system from the foot-and-mouth disease virus (FMDV) to *T. reesei* to express a readily screenable marker protein that is co-translated with a target protein. The 2A peptide sequence allows multiple independent genes to be transcribed as a single mRNA. Upon translation, the 2A peptide sequence causes a “ribosomal skip” generating two (or more) independent gene products. When the 2A peptide is translated, the “skip” occurs between its two *C*-terminal amino acids (glycine and proline), resulting in the addition of extra amino acids on the *C* terminus of the upstream protein and a single proline addition to the *N* terminus of the downstream protein. To test this approach, we have cloned two heterologous proteins on either side of a modified 2A peptide, a secreted cellobiohydrolase enzyme (Cel7A from *Penicillium funiculosum*) as our target protein, and an intracellular enhanced green fluorescent protein (eGFP) as our marker protein. Using straightforward monitoring of eGFP expression, we have shown that we can efficiently monitor the expression of the target Cel7A protein.

**Results:**

Co-expression of Cel7A and eGFP via the FMDV 2A peptide sequence resulted in successful expression of both test proteins in *T. reesei*. Separation of these two polypeptides via the modified 2A peptide was ~100% efficient. The Cel7A was efficiently secreted, whereas the eGFP remained intracellular. Both proteins were expressed when cloned in either order, i.e., Cel7A-2A-eGFP (C2G) or eGFP-2A-Cel7A (G2C); however, eGFP expression and/or functionality were dependent upon the order of transcription. Specifically, expression of Cel7A was linked to eGFP expression in the C2G orientation, whereas expression of Cel7A could not be reliably correlated to eGFP fluorescence in the G2C construct. Whereas eGFP stability and/or fluorescence were affected by gene order, Cel7A was expressed, secreted, and exhibited the expected functionality in both the G2C and C2G orientations.

**Conclusions:**

We have successfully demonstrated that two structurally unrelated proteins can be expressed in *T. reesei* using the FMDV 2A peptide approach; however, the order of the genes can be important. The addition of a single proline to the *N* terminus of eGFP in the C2G orientation did not appear to affect fluorescence, which correlated well with Cel7A expression. The addition of 21 amino acids to the *C* terminus of eGFP in the G2C orientation, however, appeared to severely reduce fluorescence and/or stability, which could not be linked with Cel7A expression. The molecular biology tool that we have implemented in this study will provide an efficient strategy to test the expression of heterologous proteins in *T. reesei*, while also providing a novel platform for developing this fungus as an efficient multi-protein-expressing host using a single polycistronic gene expression cassette. An additional advantage of this system is that the co-expressed proteins can be theoretically produced at equimolar ratios, as (A) they all originate from a single transcript and (B) unlike internal ribosome entry site (IRES)-mediated polycistronic expression, each cistron should be translated equimolarly as there is no ribosomal dissociation or reloading between cistrons.

**Electronic supplementary material:**

The online version of this article (doi:10.1186/s13068-017-0710-7) contains supplementary material, which is available to authorized users.

## Background

*Trichoderma reesei* is well known for its ability to secrete cellulases at very high levels. Up to 100 g/L native cellulases have been reported from mutated strains [1, 2]. There have been several advances made with respect to improving *T. reesei* strains for expression of native cellulase enzymes [3–6]. These include the development of an efficient transformation system, varied selection markers, and strong promoters for expression of cellulases [7]. However, the use of *T. reesei* as a heterologous expression host has been limited to only a few select proteins (reviewed in [7]). Moreover, even successful protein-expressing strains often have very low yields.

Several factors are considered important for expression of foreign genes in filamentous fungi, such as *T. reesei*, including transformation efficiency of the strain, codon bias, promoter strength, gene silencing effects, GC content of the gene, site of integration, copy number, and correct post-translational processing of heterologous proteins. Among these, the foremost consideration is the ability to transform the strain and identify positive transformants from a pool of colonies. A transformant can be typically identified by growth on selective media; however, mere growth on selection media does not guarantee the expression of the gene of interest (GOI) nor can it be used to predict the level of expression. For example, fragmentation or rearrangement of the plasmid construct prior to integration may lead to separation of the selectable marker from the GOI. As a consequence, additional techniques, such as PCR-based screening of individual transformants using gene-specific primers, western blotting with specific antibodies to identify the protein of interest, or activity assays must be carried out in order to verify the presence of the GOI. Whereas these techniques are currently being used in *T. reesei* research, they are tedious when screening hundreds of colonies to find the best expressing transformant. Depending on copy number, its site(s) of integration, and the nature of the gene itself, the level of expression may be extremely low or essentially zero. The former two aspects can be addressed by targeted transformation into mutants lacking the non-homologous end-joining (NHEJ) pathway [8–11]. Using NHEJ mutant host strains, heterologous genes can be targeted into genomic locations where high expressing genes are typically present. Another commonly used strategy to track expressibility of a GOI is to fuse it to a trackable gene (such as a fluorescent, antibiotic, or auxotrophic marker) or a highly expressible native gene. However, such fusions may lead to alteration or even complete loss of function of the target or marker protein. Despite the availability of a few laborious (genomic DNA extraction, western blotting) and less reliable (colony PCR) techniques to identify the presence and expression of GOI, a simple, reliable, and robust way to ensure independent GOI expression (i.e, without fusion) as well as identifying a positive GOI-expressing transformant from the pool of transformants is still lacking.

In theory, the use of viral 2A peptides ensures a 1:1 expression ratio of the two linked genes. The 2A peptides are found in many viruses, notably positive-strand RNA viruses, such as picornaviridae [12, 13]. The foot-and-mouth disease virus (FMDV) genomes (prototypical *Aphthovirus* genus) contain a single, long, open reading frame that encodes a polyprotein of ~225 kDa. However, the full-length translation product is seldom found due to rapid “primary” discontinuous polyprotein translation occurring at the *C*-terminal end of the 2A peptide sequence found within this ORF [13]. The FMDV 2A peptide contains a specific 19 amino acid sequence that “auto-separates” the viral polyproteins during translation [14–16]. The mechanism of separation of the polyproteins involves hydrolysis of the nascent polypeptide from the tRNA during ribosomal translation of the 2A peptide sequence, wherein the peptidyl (2A)-tRNA^Gly^ ester linkage to the glycyl-tRNA in the ribosomal P-site is hydrolyzed, thereby releasing the nascent polypeptide from the ribosomal translational complex. Continued synthesis of the downstream protein sequence starting with the sequential proline on the *C* terminus of the 2A peptide sequence still occurs [15]. It appears that the downstream polypeptide is never directly bound to the upstream protein through a peptide bond. Instead, the 2A sequence induces hydrolysis of the upstream protein 2A glycine (position 18 in native FMDV 2A) from its tRNA. The position 19 proline tRNA still complexes with the ribosome but instead of being linked to the 18-glycine, it is used as the *N* terminus of a new polypeptide. Two independent proteins are thus produced due to the lack of formation of a peptide bond during the translation process, also referred to as “ribosomal-skip” mechanism [15]. Referencing the 2A peptide as “self-cleaving” is somewhat of a mischaracterization, as the two translated sequences are never covalently linked. This mechanism of discontinuous protein translation has been shown to be specific to eukaryotic 80S ribosomes only (not to 70S prokaryotic ribosomes). Donnelly et al. [12] also observed ~90% cleavage activity with a 19 amino acid 2A peptide sequence, which was further enhanced to ~96% by the addition of as few as five amino acids to the *N*-terminal end of the 2A peptide [14]. Further addition of amino acids (up to 39 amino acids) to the *N*-terminal end resulted in increasing cleavage efficiency to ~100% [14].

The advantage of the 2A peptide system is that two or more independent proteins can be produced at near-stoichiometric levels unlike internal ribosome entry site (IRES)-mediated polycistronic expression where ribosomes are independently recruited at distinct regions with the mRNA [14, 15, 17, 18]. Up to nine proteins have been linked and successfully co-translated and separated with 2A sequences in a methylotrophic yeast, *Pichia pastoris* [19]. With respect to *T. reesei*, the 2A peptide approach could drastically reduce the time for screening of high GOI-expressing transformants if a simple marker protein was included. Here, we report successful co-expression of two independent heterologous proteins in *T. reesei*; cellobiohydrolase Cel7A from *Penicillium funiculosum* and a fluorescent marker protein eGFP, separated by a 22 amino acid modified FMDV 2A peptide sequence. Both proteins are produced as separate polypeptides from a single transcript with an observed 2A separation efficiency of ~100% based on western blot analysis. Availability of this molecular tool will dramatically improve the transformation screening efficiency and further develop *T. reesei* as a robust protein expression host, where multiple proteins can be expressed simultaneously for both academic and industrial pursuits. Aside from co-expression of various industrial enzymes, this tool could be exploited for expression of complete metabolic pathways to generate similar stoichiometric levels of proteins for bioproducts synthesis from *T. reesei*.

## Results and discussion

An efficient way to identify and track transformants expressing heterologous and homologous genes has been lacking in eukaryotic organisms, such as *T. reesei*. Although expressing colonies can be identified by intensively screening the transformants by western blot analysis or protein activity assays, this is extremely difficult for proteins lacking specific antibodies or with no functional activity assays. Here we have presented a simple and efficient way of detecting successful expression of genes (foreign or native) using a 2A peptide-driven approach.

### Transformation of FMDV 2A peptide in *T. reesei*

**Fig. 1:**
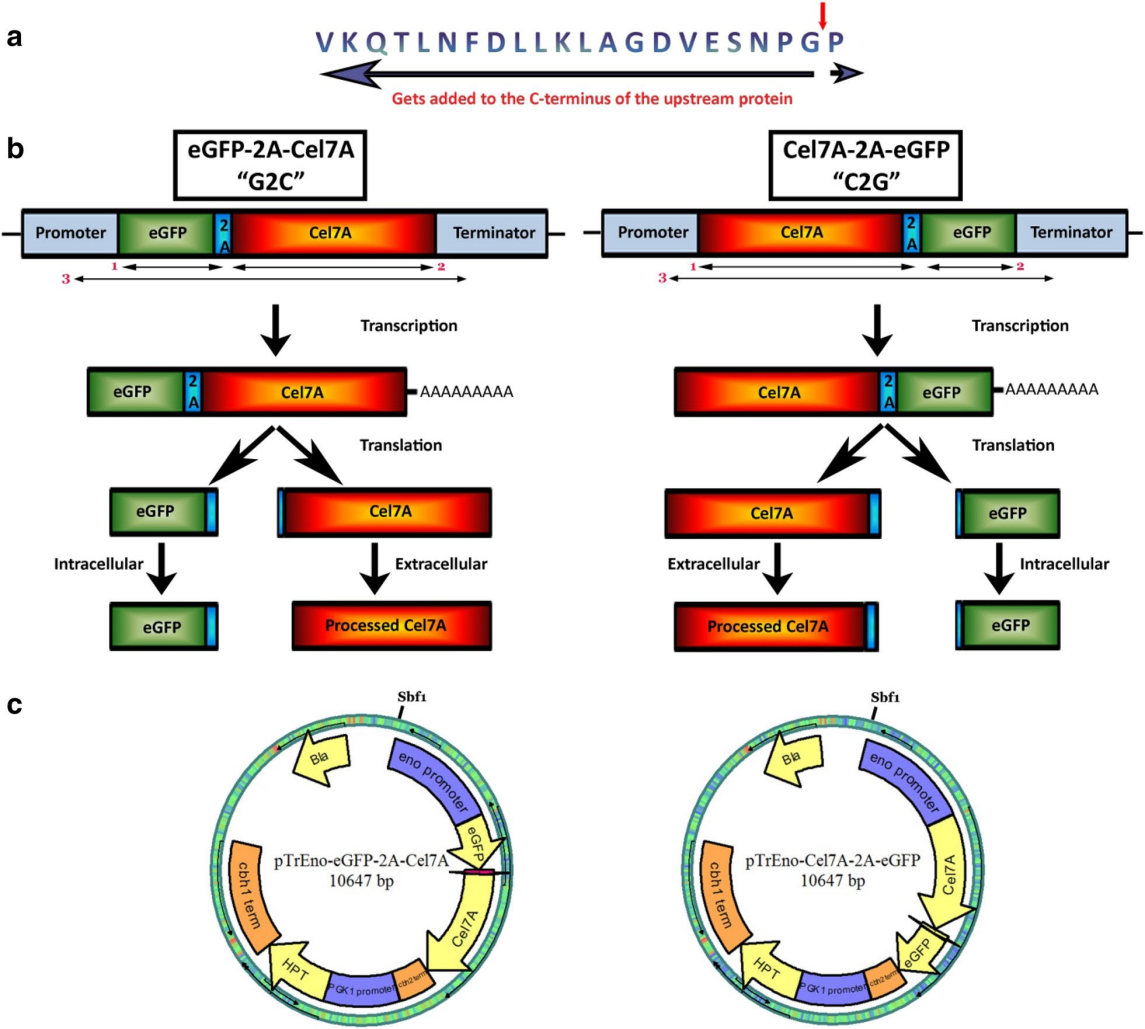
Constructs used in this study. **a** Modified FMDV 2A peptide sequence used in this study. *Red arrow* represents the site of ribosomal skipping. The *long blue arrow* represents the peptide that gets added to the *C*-terminal end of the upstream protein. The *short blue arrow* represents the amino acid (proline) that gets added to the *N*-terminal end of the downstream protein. **b** Bicistronic gene constructs for testing expression of Cel7A and eGFP protein in *T. reesei*. Cel7A and eGFP are separated by FMDV 2A peptide sequence in either orientation (*left panel* and *right panel*). Upon transcription of the bicistronic gene construct, a single mRNA transcript containing eGFP and Cel7A are produced, which upon translation gets cleaved after the glycine residue within the 2A peptide to yield two independent proteins. 21 amino acids of the 2A peptide get added to the protein upstream of the peptide and one amino acid (proline) gets added to the downstream protein. *Horizontal arrows* and the numbers *1*, *2*, and *3*, below the gene cassettes, represent PCR products that were used to confirm the presence of individual genes in the transformants. **c** Plasmid maps of the two constructs used in this study. The restriction site, *Sbf*I, represents the site of linearization of the plasmid. *Eno* enolase, *PGK1* phosphoglycerate kinase, *HPT* hygromycin phosphotransferase, *term* terminator

We generated two different plasmids using *p*TrEno (hygromycin resistance marker and strong constitutive strong *eno* promoter) as the starting vector such that the eGFP and Cel7A genes were on either sides of the modified 2A peptide sequence [14–16]. The two vectors contained the same genes in inverse order; Cel7A-2A-eGFP (C2G) or eGFP-2A-Cel7A (G2C). The constructs, when successfully expressed, should result in two proteins separated between the glycine 21 and proline 22 within the 2A peptide sequence, with the protein downstream of the 2A peptide containing an extra proline at the *N* terminus and the upstream protein containing an additional 21 amino acids at its *C*-terminal end. The expression cassette is as shown in Fig. 1. Transformation of both these constructs resulted in hygromycin-resistant colonies. As a first step toward predicting Cel7A protein expression via co-expression of eGFP, we tested 22 hyg^r^ colonies for eGFP expression based on fluorescence using a Safe Imager 2.0 blue light transilluminator (470 nm, ThermoFisher Scientific Inc., Grand Island, NY, USA). Although, there was noticeable visual difference between the fluorescence levels in these micro-cultures (data not shown), it was difficult to identify actual eGFP-expressing colonies from the pool of transformants using this instrument due to low-level background fluorescence of *T. reesei*.

### Confirmation of efficient cleavage of 2A peptide in *T. reesei*

**Fig. 2:**
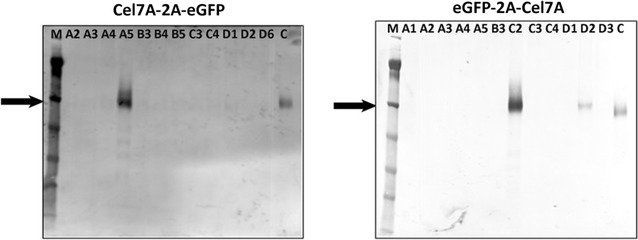
Screening of transformants for identification of Cel7A-expressing colonies. Individual transformant colonies arising from the two different plasmid constructs (cel7A-2A-eGFP and eGFP-2A-Cel7A) were transferred to liquid medium containing hygromycin and grown for 3 days at 30 °C. Western blotting of secretome was performed for a few of the selected colonies from the 24-well plates using anti-Cel7A antibody. *Lanes*. *M* molecular weight marker, *A*, *B*, *C*, and *D* followed by numbers represent well coordinates, *C* Cel7A control

Since it was difficult to positively identify eGFP-expressing clones under blue light (background fluorescence), we identified positive Cel7A-expressing clones by western blotting. Our analysis revealed that Cel7A-expressing clones were detected with a very low frequency. Only two of 12 transformants screened showed detectable Cel7A expression, to varying degrees, among each of the C2G and G2C transformants (Fig. 2). Among the C2G transformants, colonies A4 and A5 showed Cel7A expression, with A5 being the best expressing clone (Fig. 2, left panel). Expression of Cel7A in colony A4 was extremely low. Among the G2C transformants, colonies C2 and D2 showed Cel7A expression with C2 expressing better than the D2 clone. This observation highlighted the low efficiency and high variability of protein expression in this eukaryotic system, even in hygromycin-resistant transformants. The estimated molecular weight of Cel7A, as detected in the western blot, was ~53 kDa, which is the expected size of intact Cel7A (Fig. 2). If the 2A peptide had not efficiently separated, the apparent molecular weight would have been ~82 kDa (52.5 kDa for Cel7A + 2.3 kDa for 2A + 26.9 kDa for eGFP). This demonstrated that the 2A peptide was effective in both gene arrangements.

**Fig. 3:**
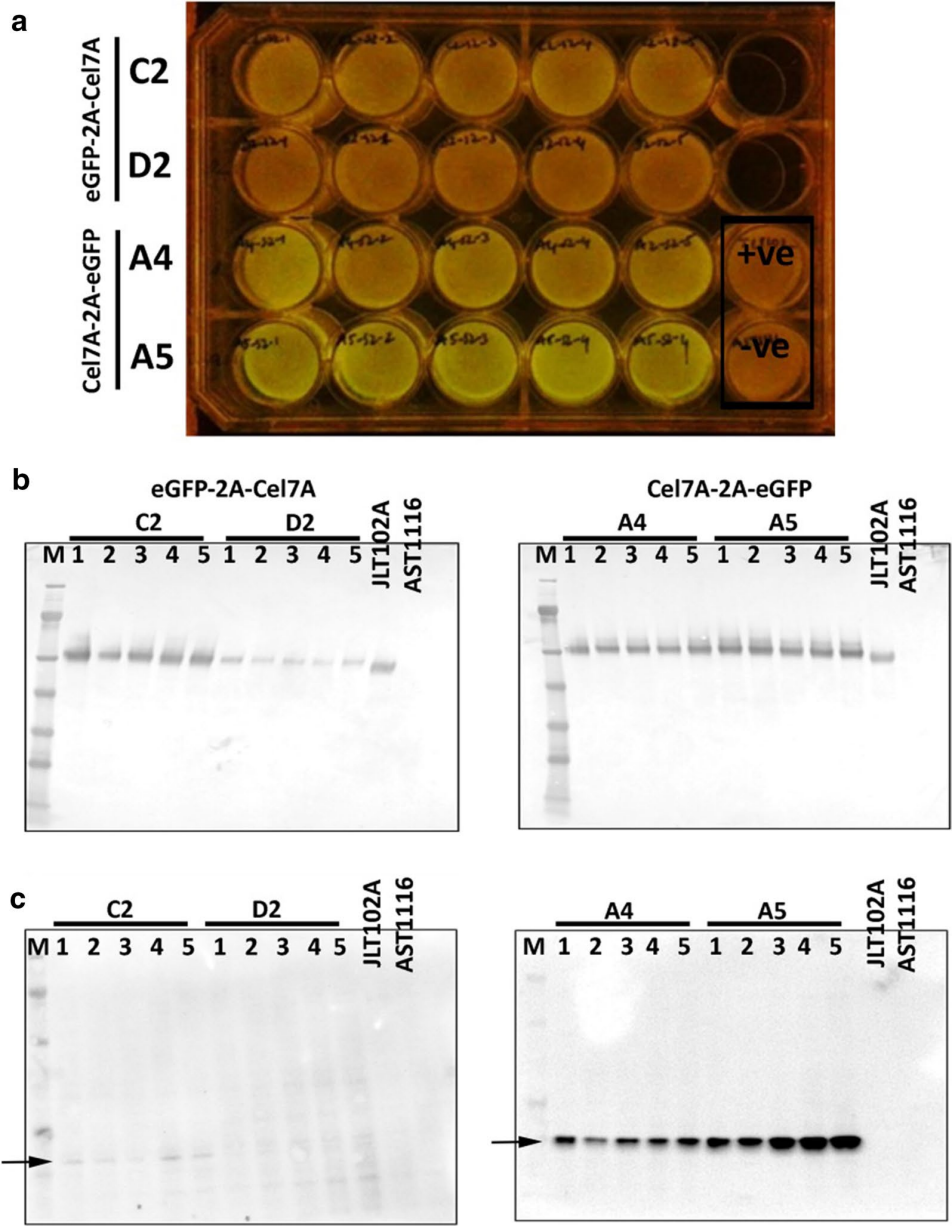
Demonstration of efficient cleavage of the FMDV 2A peptide in *T. reesei*. Clonal isolates of the two FMDV 2A-based constructs were compared for their eGFP fluorescence and for their efficiency in expressing Cel7A protein. **a** Individual clonal isolates were transferred to liquid media and incubated for 3 days at 30 °C in 24-well culture plates. eGFP fluorescence was visualized under *blue light*. Each of the *rows* represents five independent clones from a single Cel7A-expressing transformant. +ve and –ve indicate wells containing Cel7A-expressing and Cel7A-deleted transformants, respectively. **b** Western blotting of secretome from individual wells using anti-Cel7A antibody. **c** Western blotting of intracellular protein extract from individual clonal isolates using anti-eGFP antibody. *Lanes M* marker, *1*, *2*, *3*, *4*, *5*, correspond to individual well coordinates, C2, D2, A4, and A5, represent original Cel7A-expressing transformants from which clonal isolates were generated, JLT102A (AST1116 expressing native Cel7A under the *eno* promoter), AST1116 (Cel7A-deleted *T. reesei* QM6a strain)

In order to determine the consistency of fluorescence to Cel7A expression, we generated five clonal isolates for two transformants that showed varying degrees of Cel7A expression; as well as visual eGFP fluorescence from each of the 2A orientation cassettes. The selected C2G transformants were A4 (very low fluorescence) and A5 (very high fluorescence) and C2 (high fluorescence) and D2 (medium fluorescence) for the G2C transformants (Fig. 2). Clonal isolates were obtained by restreaking a spore suspension obtained from each of the four positive Cel7A transformants onto hygromycin selection plates. Five individual colonies arising from this restreak were inoculated into MAG medium containing hygromycin. We observed that these clonal isolates clearly displayed consistent degrees of fluorescence based on blue light visualization (Fig. 3a) that represented the varied expression levels in the parental strains. Whereas A4 and A5 clones displayed better fluorescence, C2 and D2 clones showed poor fluorescence (Fig. 3a). Western blotting was performed on cell-free media, which showed that in the C2G orientation, A4 and A5 clones showed relatively high levels of Cel7A protein (Fig. 3b, right panel). On the other hand, in the G2C orientation, C2 clones showed higher Cel7A protein expression than D2 (Fig. 3b, left panel). In order to confirm if this result correlated with eGFP protein expression, we performed western blotting of intracellular protein extract using anti-eGFP antibody. It should be noted that no detectable eGFP expression was detected extracellularly in any of these clonal isolates via visual fluorescence observation or western blot analysis (data not shown). We observed that eGFP protein was expressed at relatively similar levels in the C2G strains A4 and A5—that is low levels in A4 and high levels in A5 (Fig. 3c, right panel). In contrast, eGFP was barely detectable in the G2C strains. Specifically, C2 showed very low eGFP levels and D2 did not show eGFP bands at the expected molecular weight (Fig. 3b, left panel). These observations highlight that the arrangement of the two genes can be critical in using 2A systems, wherein the 2A remnants can have potential deleterious effects on the target proteins. A promising observation is that both Cel7A and the eGFP proteins were expressed at the expected molecular weight (i.e., ~53 kDa for Cel7A and ~27 kDa for eGFP). No detectable expression of an uncleaved protein product at ~82 kDa was observed in the western blots. This suggested that the 2A peptide was being cleaved at ~100% efficiency and was resulting in the generation of the two complete gene products, Cel7A and eGFP in *T. reesei*.

### Cel7A is functional in either sequence

**Fig. 4:**
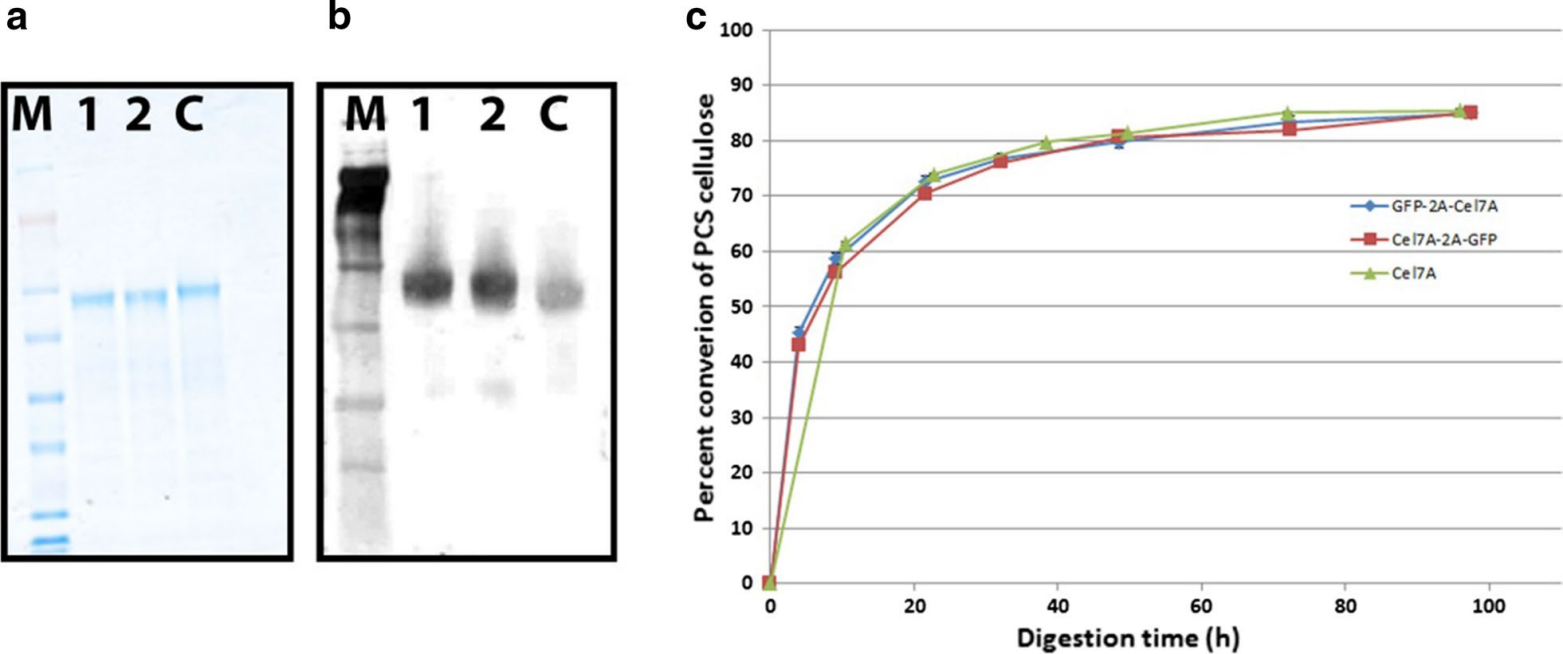
Cel7A protein purification and enzyme activity of the purified protein on pretreated corn stover (PCS) as a substrate. **a** Coomassie-stained SDS-PAGE gel showing a single band of ~53 kDa purified Cel7A protein. **b** A parallel SDS-PAGE gel was used to identify the purified proteins by western blotting using anti-Cel7A antibody. **c** Cellulase enzyme activity of Cel7A proteins obtained from expression of the two different versions of the 2A cassette (i.e., G2C and C2G), compared to purified *P. funiculosum* Cel7A, shows similar conversion pattern. *M* molecular weight marker, *Lanes 1* and *2* represent purified Cel7A proteins from G2C and C2G cassettes, respectively. *Lane C* represents purified *P. funiculosum* Cel7A

Once we observed that eGFP stability/fluorescence was affected by its position within the 2A cassette, we questioned whether or not the activity of Cel7A was also affected in a similar manner. To answer this question, we purified Cel7A proteins to homogeneity from either sequence using extensive Fast Protein Liquid Chromatography (FPLC). The specific clones used for purification were SV001 (eGFP-2A-Cel7A #C2-1, Fig. 3a) and SV004 (Cel7A-2A-eGFP #A5-4, Fig. 3a). The purified proteins were observed as single bands of ~53 kDa on SDS-PAGE (Fig. 4a, b). We performed in vitro biomass hydrolysis assays of dilute acid-pretreated corn stover using these purified Cel7A proteins plus standard endocellulase and β-d-glucosidase to quantify the cellulose conversion efficiency of Cel7A obtained from these two gene orientations. Our analysis showed that Cel7A obtained from both orientations displayed similar conversion profiles (Fig. 4c). The rate of cellulose conversion activity of these two Cel7A variants was comparable to the rate of conversion of native *P. funiculosum* Cel7A (Fig. 4c). As the Cel7A contains a secretion signal peptide sequence that gets cleaved during secretion of the processed protein, the resultant final protein from the G2C is the same as the native sequence. The addition of a single proline to the *N* terminus of the Cel7A signal sequence does not appear to negatively affect secretion. In the C2G, Cel7A activity also remained unaffected, suggesting that the expressed protein was able to undergo proper folding and retained a conformation very close to that of the native protein. It is especially telling that activity was measured on an insoluble lignocellulosic substrate, indicating that the *C*-terminal cellulose-binding domain of Cel7A is fully functional, despite the extra 21 amino acids added *C*-terminally. This retained functionality is indicative that other *C*-terminal tags may be used to monitor Cel7A.

### Expression of Cel7A can be correlated to eGFP fluorescence when expressed in the C2G orientation

**Fig. 5:**
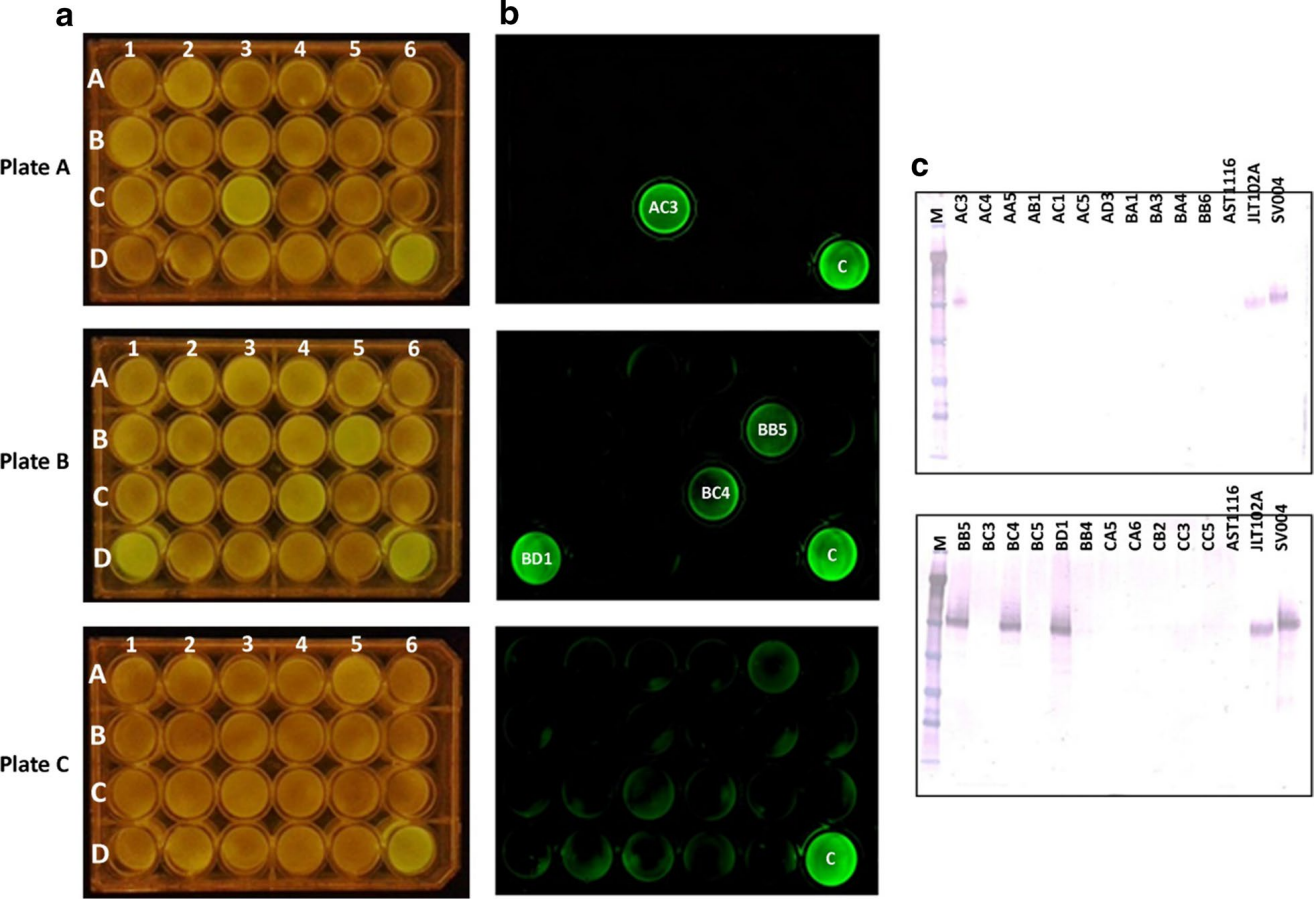
Correlation between fluorescence intensity and Cel7A expression in the C2G orientation. Individual transformants were transferred to three 24-well plates containing MAG medium supplemented with hygromycin and allowed to grow for 3 days at 30 °C. Rows are labeled as *A*–*D*. Columns are labeled as *1–6*. Plates are labeled as plate A, plate B, and plate C. **a** Mycelia-containing plates visualized under *blue light*. **b** The same plates were also visualized using FluorChem Q imaging system (Cell Biosciences, Santa Clara, CA, USA). **c** Western blotting on selected colonies using anti-Cel7A antibody. Each *lane* represents secretome obtained from one well of the 24-well plate. *M* marker, *letters* followed by *numbers* represent coordinates on the 24-well plates. The *first letter* represents the plate ID, the *second letter* represents the *row*, and *numbers* following the *two letters* represent the *column number*. Coordinates D4, D5, and D6 in each plate represent the control strains, AST1116 (Cel7A-deleted *T. reesei* QM6a strain), JLT102A (AST1116 expressing native Cel7A under the *eno* promoter), and SV004 (Cel7A-2A-eGFP cassette containing very high eGFP fluorescence strain; colony A5-4 in Fig. 3a)

**Fig. 6:**
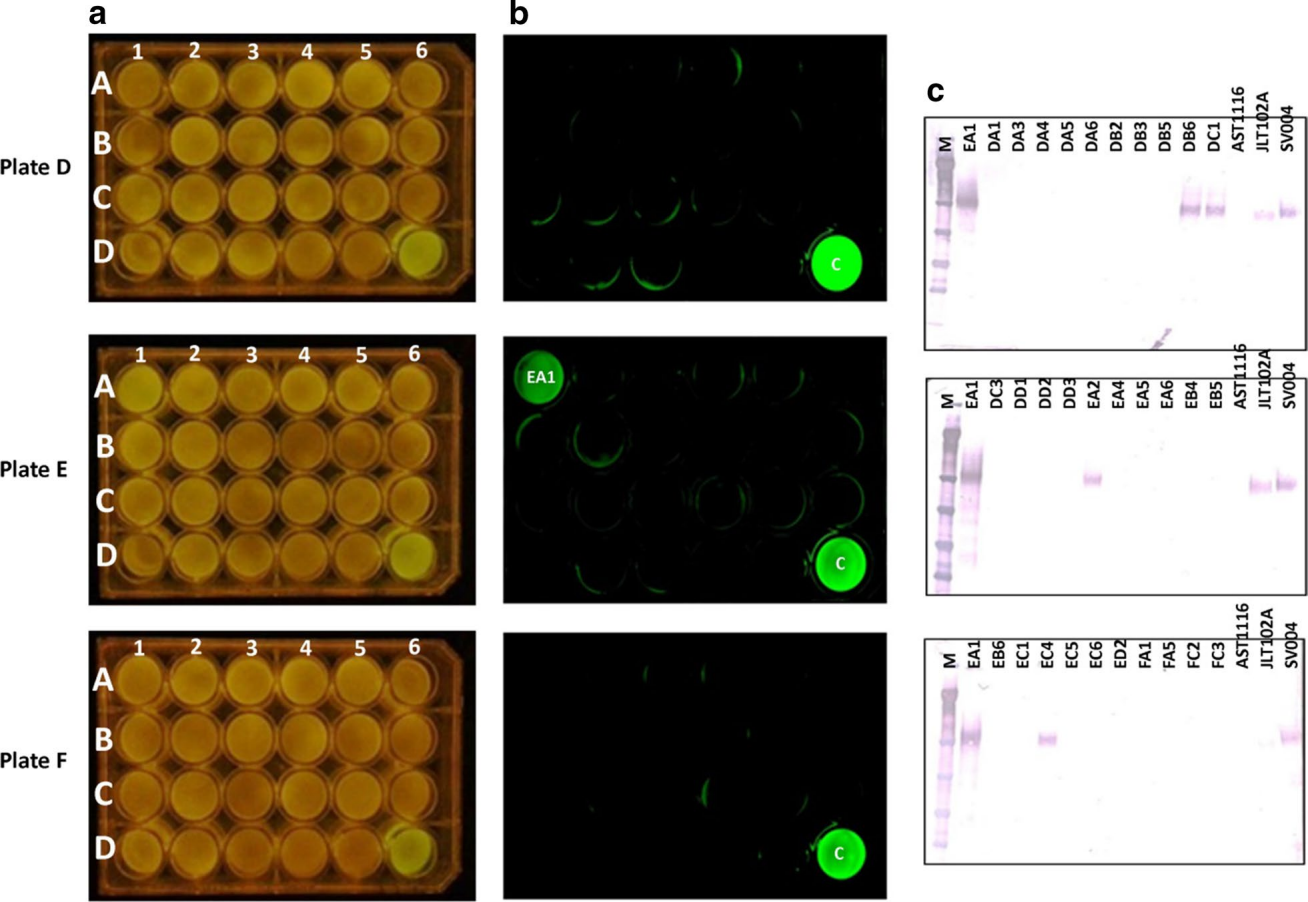
Correlation between fluorescence intensity and Cel7A expression in the G2C orientation. Individual transformants were transferred to three 24-well plates containing MAG medium supplemented with hygromycin and allowed to grow for 3 days at 30 °C. Rows are labeled as *A*–*D*. Columns are labeled as *1*–*6*. Plates are labeled as plate A, plate B, and plate C. **a** Mycelia-containing plates visualized under *blue light*. **b** The same plates were also visualized using FluorChem Q imaging system (Cell Biosciences, Santa Clara, CA, USA). **c** Western blotting on selected colonies using anti-Cel7A antibody. Each *lane* represents secretome obtained from one well of the 24-well plate. *M* marker, *Alphabets* followed by *numbers* represent *coordinates* on the 24-well plates. The *first alphabet* represents the *plate ID*, the *second alphabet* represents the *row*, and *numbers* following the *two alphabets* represent the *column number*. Coordinates D4, D5, and D6 in each plate represent the control strains, AST1116 (Cel7A-deleted strain), JLT102A (Cel7A-expressing strain in a Cel7A null background), and SV004 (Cel7A-2A-eGFP cassette containing very high eGFP fluorescence strain; colony A5-4 in Fig. 3a)

**Fig. 7:**
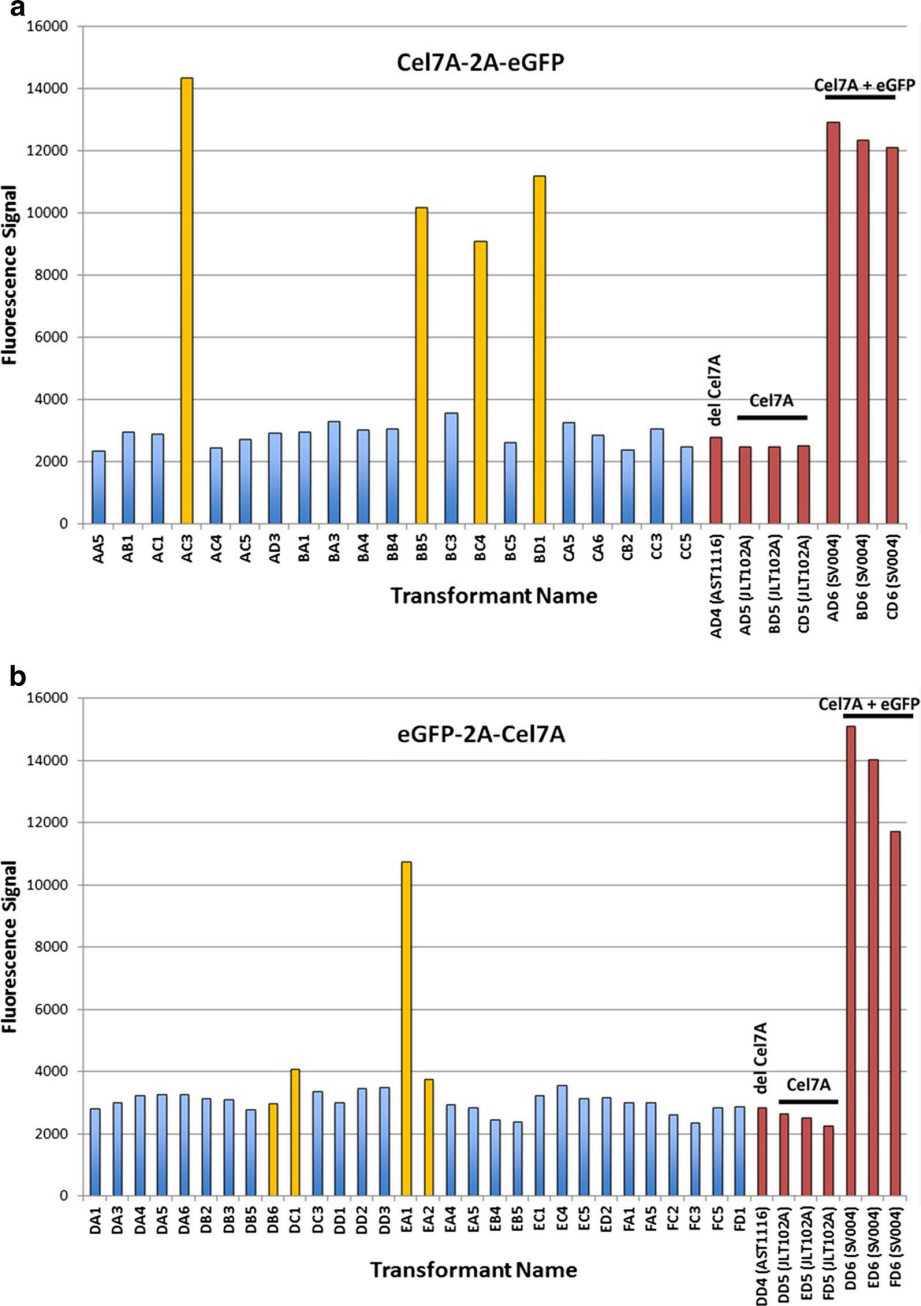
Fluorescence signal intensity of individual transformants and correlation with expression of Cel7A by western blotting. **a** Transformants for Cel7A-2A-eGFP were analyzed for fluorescence intensity and Cel7A expression. **b** Transformants for eGFP-2A-Cel7A were analyzed for fluorescence intensity and Cel7A expression. Fluorescence intensity was measured using FLUOstar Omega plate reader (BMG Labtech GmbH) and is represented using arbitrary fluorescence units. Western blotting was performed on cell-free media as shown in Figs. 4 and 5. Each *bar* represents data obtained for a single transformant colony. *Blue bars* indicate transformants showing no Cel7A expression. *Yellow bars* indicate transformants showing Cel7a expression. *Red bars* represent control strains. Controls: JLT102A (AST1116 expressing native Cel7A under the *eno* promoter), AST1116 (Cel7A-deleted *T. reesei* QM6a strain), and SV004 (AST1116 expressing Cel7A-2A-eGFP; colony A5-4 in Fig. 3a)

The purpose of this approach was to use eGFP as a marker for monitoring expression of proteins, such as Cel7A (in this study); as well as other difficult to monitor heterologous proteins in future *T. reesei* studies. Initially, it proved to be difficult to monitor eGFP expression with simple blue light fluorescence measurement. Therefore, we decided to quantify the eGFP fluorescence using a FLUOstar Omega plate reader (BMG Labtech GmbH, Ortenburg, Germany) and compare fluorescence units (FLU) to the level of Cel7A expression by western blotting. This was carried out by transferring 63 individual colonies of each gene orientation from transformation plates into liquid medium containing hygromycin (100 µg/mL) in 24-well plates. After allowing growth of the mycelial mat, fluorescence measurement was performed using the plate reader (Figs. 5, 6, 7). Simultaneously, we also used a FluorChem Q imaging system (Cell Biosciences, Santa Clara, CA, USA) to visually differentiate fluorescent colonies from the low or non-fluorescent colonies (Figs. 5b, 6b). We observed that the fluorescent signal measurements ranged from ~3000 to ~15000 FLUs in the different wells (Fig. 7), with relatively few at the upper end (>9000) and the majority at ~4000 or less. We did not observe any “medium” fluorescence colonies. This may be due to the “enhanced” nature of the eGFP providing high fluorescence even at lower levels or, more simply, that the three days growth provided enough time for positive expressing colonies to saturate themselves with eGFP regardless of expression levels. We picked all the high fluorescence wells (four for C2G, one for G2C) and numerous random representatives of low-fluorescence colonies for each group to confirm the expression of Cel7A by western blotting (Figs. 5c, 6c). We observed that the low fluorescent colonies did not show expression of Cel7A in the C2G orientation (Figs. 5, 7a). However those colonies showing fluorescence of over 9000 FLUs showed high levels of Cel7A expression in this orientation (Figs. 5, 7a).

We further observed that in the C2G orientation, expression of eGFP was directly related to the expression of Cel7A (Fig. 5). All the colonies that showed fluorescence on the plate (Fig. 5a, b) also showed expression of Cel7A in the western blot analysis (Fig. 5c). In contrast, this was not the case with the G2C orientation. In the G2C population, the low fluorescent colonies varied in Cel7A expression (Figs. 6, 7b). A few of them, such as DB6, DC1, DD5, EA2, and EC4, showed positive Cel7A expression (Figs. 6, 7b). As could be expected from the fluorometer results, these colonies did not show significant fluorescence under the FluorChem Q imaging system, indicating that even though they were expressing Cel7A, eGFP was not a reliable indication of Cel7A expression. The sole high fluorescent colony (EA1) showed Cel7A expression similar to the C2G orientation (Figs. 6c, 7b). These results clearly suggest that in the G2C orientation, the fluorescence measurement cannot necessarily be correlated to the expression of Cel7A protein, especially in the low fluorescent clones. While we cannot explain the single high eGFP/high Cel7A clone, the overall low fluorescence, even among the Cel7A positive clones, could be explained by the fact that in the G2C orientation, a 21 amino acid remnant of the FMDV 2A peptide gets added to the *C*-terminal end of the processed eGFP protein (after cleavage of the 2A peptide), which is not the case with the C2G orientation. We speculate that addition of 21 amino acids to the eGFP *C* terminus affects the overall stability of the protein, causes mis-folding, or interference with the fluorescence through quenching or electron transfer interference, resulting in lower levels of eGFP and/or lower fluorescence. Low anti-eGFP western blot detection suggests that degradation of eGFP from the G2C constructs is causing the discontinuity between Cel7A and eGFP protein levels in the G2C order. We cannot, however, rule out that the extra 21 amino acids on the G2C are interfering with anti-GFP antibody binding and detection. The single proline added to the *N* terminus of eGFP in the C2G orientation is much less likely to interfere with protein stability or function.

In order to determine if the lack of Cel7A or eGFP expression was due to non-insertion of gene(s) into the genome or due to fragmentation of the expression cassette, we performed genomic PCR analysis on colonies generated from an independent transformation experiment from both the gene cassettes (Tables 1, 2). The specific gene products used to confirm the presence of *cel7A*, *eGFP,* or both within the cassette are shown in Fig. 1b. The primers used in this analysis are shown in Additional file 1. We observed that all colonies that showed Cel7A expression to detectable levels (Tables 1, 2; Additional files 2 and 3), irrespective of their fluorescent intensities, show the presence of both *cel7a* and *eGFP* genes (Tables 1, 2; Additional files 3 and 4). Colony A2 in the C2G orientation, which did not show detectable levels of either Cel7A or eGFP, showed the presence of only *eGFP* by PCR analysis (Table 1; Additional files 2 and 4). This demonstrated an instance where insertion of eGFP and hygromycin into the genome, albeit without eGFP expression. On the other hand, colony A4 in the C2G orientation showed no Cel7A or eGFP expression although the presence of the entire gene cassette was confirmed by PCR analysis (Table 1; Additional files 2 and 4), suggesting that the location of insertion most likely resulted in lack of expression of the gene cassette. Colony B1 in the G2C orientation showed weakly positive PCR signals for both *cel7A* and *eGFP* and absolutely no expression of either gene products (Table 2; Additional files 3 and 5), suggesting a possible copy number effect. A more striking observation was that despite having high levels of Cel7A in six of the eight G2C transformants (Table 2; Additional file 3), eGFP expression was extremely low in these transformants suggesting that the stability or fluorescence of eGFP protein was particularly affected in this orientation, likely due to the addition of the C-terminal 21 aa tag. Moreover, even the best Cel7A-expressing transformant in this orientation (C6 and D1, Table 2; Additional file 3) showed lower fluorescence and lower eGFP, in comparison to the best Cel7A-expressing transformants C2 and C5 from the C2G orientation (Table 1; Additional file 2).

**Table 1:** PCR analysis of selected C2G transformants along with their protein expression and fluorescence intensity measurements

Transformants	Fluorescence intensity	Western blot analysis		PCR analysis		
		Cel7A	eGFP	PCR-1	PCR-2	PCR-3
A1	8393	+	+	+	+	+
C2	16,062	+	+	+	+	+
C4	2462	+	+	+	+	+
C5	17,975	+	+	+	+	+
D1	4358	+	+	+	+	+
D3	7360	+	+	+	+	+
A2	1976	−	−	−	+	−
A4	2736	−	−	+	+	+
AST1116	2130	−	−	−	–	−
JLT102	2088	+	−	−	–	−
SV001	ND	+	+	−	+	+
SV002	ND	+	+	−	+	+
SV004	10,539	+	+	+	+	+

Fluorescent intensities are shown in arbitrary fluorescence units (FLU)

+ and − under western blot and PCR analysis columns represent presence or absence of the protein and gene, respectively, *Faint* represents very low, but detectable levels of the respective protein, *ND* denotes no data available, *Controls* JLT102A (AST1116 expressing native Cel7A under the *eno* promoter), AST1116 (Cel7A-deleted *T. reesei* QM6a strain), SV001 and SV002 (AST1116 expressing eGFP-2A-Cel7A; colonies C2-1 and D2-1 in Fig. 3a), and SV004 (AST1116 expressing Cel7A-2A-eGFP; colony A5-4 in Fig. 3a)

**Table 2:** PCR analysis of selected G2C transformants along with their protein expression and fluorescence intensity measurements

Transformants	Fluorescence Intensity	Western blot analysis results		PCR analysis results		
		Cel7A	eGFP	PCR-1	PCR-2	PCR-3
C6	6193	+	+	+	+	+
D1	6837	+	+	+	+	+
C2	4194	+	Faint	+	+	+
A1	4444	+	Faint	+	+	+
A2	3684	+	+	+	+	+
C3	3822	+	+	+	+	+
C4	2099	Faint	−	+	+	+
B1	2352	−	−	+	+	−
AST1116	2032	−	−	−	−	−
JLT102	2204	+	−	+	−	−
SV001	ND	+	+	+	+	+
SV002	ND	+	+	+	+	+
SV004	13892	+	+	+	+	+

Fluorescent intensities are shown in arbitrary fluorescence units (FLU)

Controls: JLT102A (AST1116 expressing native Cel7A under the *eno* promoter), AST1116 (Cel7A-deleted *T. reesei* QM6a strain), SV001 and SV002 (AST1116 expressing eGFP-2A-Cel7A; colonies C2-1 and D2-1 in Fig. 3a), SV004 (AST1116 expressing Cel7A-2A-eGFP; colony A5-4 in Fig. 3a)

+ and − under western blot and PCR analysis columns represent presence or absence of the protein and gene, respectively, *Faint* represents very low, but detectable levels of the respective protein, *ND* denotes no data available

It is known that some heterologous proteins do not express well in *T. reesei* unless fused to a native “leader” protein domain. We did not express eGFP as a single gene construct in order to confirm that it is expressible in *T. reesei*, and the fluorescence measured in the C2G orientation could be interpreted as evidence that eGFP requires a leading peptide sequence for expression in *T. reesei*; however, the high level of Cel7A present in multiple G2C constructs negates this conclusion. In order for Cel7A to be expressed in the G2C construct, the eGFP must be translated first.

Given the theoretical equimolar expression levels of 2A peptide-linked proteins, we expected to be able to correlate eGFP expression level measured by fluorescence to Cel7A expression level measured by western blot. Our intention was to identify colonies with varying levels of eGFP fluorescence and measure similarly varying levels of Cel7A. In all cases of high eGFP expression, we observed high Cel7A expression; however, the inverse, low/no eGFP and accompanying low/no Cel7A, was only consistent in the C2G case. In the other orientation (i.e., G2C), it is more stochastic, where even though eGFP fluorescence was low, Cel7a expression was still observed in some clones. This result indicates that decreased expression and/or functionality of eGFP due to added *C*-terminal amino acids to the final protein make the G2C orientation less reliable for transformant screening purposes. However, based on Cel7A activity experiments, it is clear that Cel7A activities, in both the orientations, are the same.

## Conclusions

Although *T. reesei* is known as a versatile cellulase-producing fungus and has been exploited intensively for this purpose, expressing heterologous proteins still remains a challenge in this organism. Here, we have demonstrated the use of a novel bicistronic expression platform to monitor co-expression of heterologous genes using eGFP as a marker. Our results have unequivocally demonstrated that two genes can be expressed successfully in *T. reesei* in a bicistronic manner from a single transcript using the 2A peptide approach. We have also shown that eGFP can be used as a fluorescent marker in *T. reesei* and that the levels of the transgene expression (in this case, *cbh1*) can be directly correlated to that of eGFP. We have also verified that the Cel7A activity toward pretreated corn stover is not affected by its orientation within the 2A cassette, although eGFP expression and/or stability are affected when specifically placed in the orientation that adds 21 amino acids to its *C*-terminal end (i.e., G2C orientation). We propose that this approach can be further extended to other cellulases in *T. reesei* and possibly to other transgenes, as long as addition of amino acids to the gene products’ *C* terminus does not affect their function. Furthermore, the 2A peptide approach can be expanded for expression of multiple proteins simultaneously by introducing additional 2A peptide sequences between genes. Future work in our lab will involve developing this approach toward expressing complete metabolic pathways, as well as multiple biomass conversion enzymes from a single transcript.

## Methods

### Strains and growth conditions

The *T. reesei* strains AST1116 and JLT102A used in this study were routinely maintained on Mandels Andreotti minimal medium containing 5% glucose (MAG) as described in Linger et al. [5]. For strains resistant to hygromycin, MAG medium was supplemented with 100 µg/mL of this antibiotic. AST1116 was derived from *T. reesei* (*Trichoderma reesei*) QM6a by deletion of the native *cbh1* gene [6]. JLT102A was derived from AST1116 by insertion of the native *T. reesei cbh1* gene under the control of a constitutive *eno* promoter [5].

### Plasmid construction

The vector pTrEno-PF was used as a parent vector [5]. Two different FMDV 2A peptide-based constructs were designed by Genscript (Piscataway, NJ, USA). The first one, pTrEno-Cel7A-2A-eGFP (C2G) was constructed as follows. The FMDV 2A peptide sequence and an enhanced Green Fluorescent Protein (eGFP) sequence were codon optimized and synthesized by Genscript such that the 2A and the eGFP sequences were in-frame with each other. Furthermore, an *Xba*I site was introduced at either ends of this 2A-eGFP cassette so as to introduce this cassette into the pTrEno-PF vector into the *Xba*I site. Note that neither the 2A nor the eGFP sequences included a start codon. Additionally, the stop codon was also eliminated from the Cel7A gene so as to have a contiguous Cel7A-2A-eGFP gene sequence with one start codon at the 5′ end of Cel7A gene and a stop codon at the 3′ end of eGFP coding sequence.

The second vector, pTrEno-eGFP-2A-Cel7A (G2C), was constructed as follows by Genscript. Briefly, a codon-optimized eGFP sequence along with a 2A peptide sequence was synthesized with a *Pac*I and a *Bam*HI restriction sites flanking its 5′ and 3′ ends. This eGFP-2A cassette was introduced into the pTrEno-PF vector using *Pac*I and *Bam*HI restriction sites. Note that *Bam*HI restriction site was built into the parent vector by PCR. The resulting vector now had the following gene sequence eGFP-2A-Cel7A. This contiguous gene cassette had only one start codon at the 5′ end of the eGFP gene and a stop codon at the 3′ end of the Cel7A gene.

### Transformation of plasmids into *T. reesei*

Competent spores were prepared as described in Linger et al. [5], which involved time-specific sporulation on potato dextrose agar (PDA), followed by re-sporulation, collection, and washing of spores before freezing at −80 °C as electro-competent spore stocks. 5 µg of plasmid was linearized with *Sbf*I and further purified using DNA clean and concentrator-5 kit (Zymo Research Corp, Irvine, CA). Frozen competent spores were thawed on ice and mixed with ~1.0 µg of the linearized plasmid. Electroporation was carried out using a Bio-Rad Gene Pulser (Bio-Rad Laboratories, Inc., Hercules, CA, USA) using the following conditions: 1.8 kV, 25 µF, 800 Ω, and incubated for few min on ice. 1 mL of ‘complete medium lactose’ medium was then added to the transformation mixture and transferred to six-well tissue culture plates and incubated at RT for 18 h to allow recovery and germination of spores. 200 µL of this cell suspension was then plated on PDA containing 100 µg/mL hygromycin and 0.1% (v/v) Triton X-100 for colony size restriction (PDHX) and incubated at 30 °C in lighted incubator for 2–3 days to allow colony development.

### Screening of transformants by western blotting

A small piece of mycelial fragment from transformant colonies grown on PDHX plates was transferred to 2 mL of MAG medium containing hygromycin (100 µg/mL) in a 24-well microtiter plate and incubated statically in a lighted 30 °C incubator for 3 days till a mycelial mat was observed on the liquid medium. 15 µL of cell-free culture broth (containing secreted proteins) was transferred to microcentrifuge tubes containing 5 µL SDS-PAGE loading buffer and subjected to boiling at 95 °C for 10 min. This protein extract was separated on 4–12% NuPAGE gel in MOPS buffer (200 V for 50 min). Post-separation, proteins were electro-transferred onto PVDF membrane for western blot analysis using an iBlot2 (Thermo Fisher Scientific, Inc. Grand Island, NY, USA). For hybridization of Cel7A protein, a custom generated *P. funiculosum* anti-Cel7A polyclonal antibody raised in rabbit was used as primary antibody at a dilution of 1:20,000. For detection of eGFP, anti-GFP antibody raised in mouse (Thermo Fisher Scientific Inc., Grand Island, NY, USA) was used as the primary antibody. Detection of cel7A and eGFP proteins was carried out using alkaline phosphatase-conjugated anti-rabbit secondary antibody (Thermo Fisher Scientific, Inc. Grand Island, NY, USA), and HRP-conjugated anti-mouse secondary antibody (Thermo Fisher Scientific, Inc., Grand Island, NY, USA), respectively.

### Cel7A purification

The Cel7A was purified as described earlier [5]. Briefly, fermentation broths (~8–10 L) were harvested, vacuum filtered, concentrated, and then loaded onto a 26/10 Phenyl Sepharose Fast Flow column. Buffer A was 20 mM Bis–Tris pH 6.5 and buffer B was 20 mM Bis–Tris pH 6.5, 2.0 M (NH_4_)_2_SO_4_. After binding and washing, a descending gradient of 80% B (1.6 M (NH_4_)_2_SO_4_) to 0% B over eight column volumes was used to elute the bound proteins from the column. Active fractions were identified by a *p*-nitrophenyl-β-(1→4)-d-lactopyranoside (*p*NP-L) activity assay. The *p*NP-L-active fractions were pooled and concentrated as needed. Protein was desalted and exchanged into 20 mM Bis–Tris buffer pH 6.5. This sample was then loaded onto a Tricorn 10/100 anion exchange column packed with Source 15Q and eluted with a 0–50% salt gradient over 30 column volumes. Buffers were 20 mM Bis–Tris pH 6.5 (A) and the same supplemented with 1.0 M NaCl (B). *p*NP-L activity was followed again to identify the active fractions. Active fractions were pooled, brought to 1.5 M (NH_4_)_2_SO_4_ in 20 mM Bis–Tris pH 6.5, loaded onto a Tricorn 10/100 Source-Iso column, washed, and eluted with a descending gradient from 1.6 to 0.4 M (NH_4_)_2_SO_4_. Active fractions were concentrated to <10 mL and subjected to size exclusion chromatography using a 26/60 Superdex 75 column and 20 mM sodium acetate buffer pH 5.0 containing 100 mM NaCl as the mobile phase. All chromatography buffers contained 0.02% (w/v) NaN_3_ as a microbial inhibitor. SDS-PAGE and anti-Cel7A immunoblotting were performed to assess purity.

### Fluorescence quantitation

A small mycelial fragment from a transformant colony was transferred to 24-well plates containing MAG medium (with hygromycin) and incubated for 3 days at 30 °C to allow mycelial mat formation. Following growth of the mycelial mat, plates were directly visualized in a FLUOstar Omega plate reader (BMG Labtech GmbH, Ortenburg, Germany) using excitation wavelength of 485 nm and an emission wavelength of 520 nm. Each plate included a control eGFP-Cel7A-co-expressing strain (SV004), a Cel7A-expressing strain (JLT102A), and a Cel7A-deleted strain (AST1116).

### Cel7A enzyme activity measurement

Cellobiohydrolase (Cel7A) activity was based on the conversion of the cellulose fraction of a standard dilute acid-pretreated corn stover (PCS) using a cocktail of the subject Cel7A mixed with two other standardized cellulases. Specifically, the purified Cel7A was loaded at 28 mg/g of biomass cellulose and the two ancillary enzymes, the endoglucanase *Acidothermus cellulolyticus* E1 cdY245G (Cel5A, catalytic domain, Y245G mutant) and chromatographically purified β-d-glucosidase from *Aspergillus niger*, were loaded at 1.89 and 0.5 mg/g biomass cellulose, respectively.

The Cel7A activity assay was performed essentially as described in Linger et al. [5]. Briefly, the biomass substrate, NREL dilute acid-pretreated corn stover P050921 (59.1% glucan, 25.3% lignin, 5.1% xylan), was washed with water and then with 20 mM acetic acid/sodium acetate buffer, pH 5.0, until the pH of the wash supernatant was within 0.03 units of pH 5.0 [20]. From a slurry of this washed biomass material, a series of biomass substrate aliquots were prepared in 2.0-mL high-performance liquid chromatography (HPLC) vials, in such a way that each vial contained 8.5 mg biomass cellulose. Biomass dry weights for this batch of assay vials was verified by dry weight determinations on a group of five samples co-pipetted into pre-tared vials. Adjustment of these biomass assay aliquots to a 1.7 mL final volume was done by the addition of the enzyme cocktail, which resulted in a cellulose concentration of 5.0 mg/mL in the assay mixture.

Assays were carried out in triplicate vials at 40 °C in 20 mM acetate buffer pH 5.0 containing 0.02% NaN_3_ with continuous mixing by inversion at 10 rpm while immersed in a water bath. At various times during the digestion, vials were removed from the rotator, and representative 100 μL samples containing both solids and liquid were removed from the well-mixed contents and diluted 18 folds into glass HPLC vials. The primary digestion vials were immediately resealed and returned to the rotator for continuation of the digestion process. The vials containing the samples were crimp-sealed and immersed in a boiling water bath to terminate the reaction. Quantification of released sugars was performed by high-performance liquid chromatography using a Bio-Rad HPX-87H column operated at 55 °C with 0.01 N H_2_SO_4_ as eluent at 0.6 mL/min and refractive index detection.

## Additional files

**Additional file 1.** Primers used for PCR analysis.

**Additional file 2.** Detection of Cel7A and eGFP proteins in the selected C2G transformants. A. Extracellular Cel7A detection using anti-Cel7A antibody. B. Intracellular eGFP detection using anti-eGFP antibody. Lanes M. Molecular weight marker; A1, C2, C4, C5,D1, D3, A2 and A4, transformant colonies; Ast, AST1114 (Cel7A deleted *T. reesei* QM6A strain); JLT, JLT102A (AST1116 expressing native Cel7A under the *eno* promoter); SV004, AST1116 expressing Cel7A-2A-eGFP.

**Additional file 3.** Detection of Cel7A and eGFP proteins in the selected G2C transformants. A. Extracellular Cel7A detection using anti-Cel7A antibody. B. Intracellular eGFP detection using anti-eGFP antibody. Lanes M. Molecular weight marker; C6, D1, C2, A1, A2, C3, C4 and B1, transformant colonies; Ast, AST1114 (Cel7A deleted *T. reesei* QM6A strain); JLT, JLT102A (AST1116 expressing native Cel7A under the *eno* promoter); SV004, AST1116 expressing Cel7A-2A-eGFP.

**Additional file 4.** PCR analysis to determine the presence of *cel7A*, *eGFP* and both in the C2G transformants. Lanes M. Molecular weight marker (GeneRuler 1kb DNA ladder); A1, C2, C4, C5,D1, D3, A2 and A4, C2G transformant colonies; AST1114, Cel7A deleted *T. reesei* QM6A strain); JLT102A, AST1116 expressing native Cel7A under the *eno* promoter; SV001 and SV002, AST1116 expressing eGFP-2A-Cel7A; SV004, AST1116 expressing Cel7A-2A-eGFP.

**Additional file 5.** PCR analysis to determine the presence of *cel7A*, *eGFP* and both in the G2C transformants. PCR analysis to determine the presence of *cel7A*, *eGFP* and both in the G2C transformants. Lanes M. Molecular weight marker (GeneRuler 1kb DNA ladder); C6, D1, C2, C3, A1, A2 C4 and B1, G2C transformant colonies; AST1114, Cel7A deleted *T. reesei* QM6A strain); JLT102A, AST1116 expressing native Cel7A under the *eno* promoter; SV001 and SV002, AST1116 expressing eGFP-2A-Cel7A; SV004, AST1116 expressing Cel7A-2A-eGFP.

## Abbreviations

eGFP: enhanced green fluorescent protein
Cel7A: cellobiohydrolase I
*cbh1*: cellobiohydrolase I gene
MAG: Mandels Andreotti minimal medium with 5% glucose
*p*NP-L: *p*-nitrophenyl-β-(1→4)-d-lactopyranoside
HRP: horse radish peroxidase
PDHX: potato dextrose with hygromycin and Triton X-100
PVDF: polyvinylidene difluoride
