# Supplementary material for: A versatile 2A peptide-based bicistronic protein expressing platform for the industrial cellulase producing fungus, Trichoderma reesei
Source: Biotechnol Biofuels. 2017 Feb 6;10:34. doi: 10.1186/s13068-017-0710-7 (PMC5294774; doi:10.1186/s13068-017-0710-7)
Supplement: Supplementary file 1 — Additional file 1. Primers used for PCR analysis. [file 13068_2017_710_MOESM1_ESM.docx]

| **Primer name** | **Sequence (5’ – 3’)** |
| --- | --- |
| Mf-PacI-F | ATT AAT TAA ATG TCT GCC TTG AAC TC |
| FMDV-R1 | GTT GCT CTC GAC GTC GCC |
| eGFP-F3 | GGG CGA GGA GCT CTT CAC |
| eGFP-R2 | CTT GTA CAG CTC GTC CAT GC |
| EnoIP-F1 | GCT CCT CTG GCT GGA TTT TG |
| JL383 | GAC CTG CGA CAG ACA ACC AA |
| Cel7A-BamHI-F | CCG GAT CCA TGT CTG CCT TG |
| JL387 | CCT TAC TAC TCT CAA TGT TTG TAG TAA |

Additional file 1.

Primers used for PCR analysis.
