# Supplementary material for: A versatile 2A peptide-based bicistronic protein expressing platform for the industrial cellulase producing fungus, Trichoderma reesei
Source: Biotechnol Biofuels. 2017 Feb 6;10:34. doi: 10.1186/s13068-017-0710-7 (PMC5294774; doi:10.1186/s13068-017-0710-7)
Supplement: Supplementary file 3 — Additional file 3. Detection of Cel7A and eGFP proteins in the selected G2C transformants. A. Extracellular Cel7A detection using anti-Cel7A antibody. B. Intracellular eGFP detection using anti-eGFP antibody. Lanes M. Molecular weight marker; C6, D1, C2, A1, A2, C3, C4 and B1, transformant colonies; Ast, AST1114 (Cel7A deleted T. reesei QM6A strain); JLT, JLT102A (AST1116 expressing native Cel7A under the eno promoter); SV004, AST1116 expressing Cel7A-2A-eGFP. [file 13068_2017_710_MOESM3_ESM.docx]

Additional file 3.

Detection of Cel7A and eGFP proteins in the selected G2C transformants. A. Extracellular Cel7A detection using anti-Cel7A antibody. B. Intracellular eGFP detection using anti-eGFP antibody. Lanes M. Molecular weight marker; C6, D1, C2, A1, A2, C3, C4 and B1, transformant colonies; Ast, AST1114 (Cel7A deleted *T. reesei* QM6A strain); JLT, JLT102A (AST1116 expressing native Cel7A under the *eno* promoter); SV004, AST1116 expressing Cel7A-2A-eGFP.


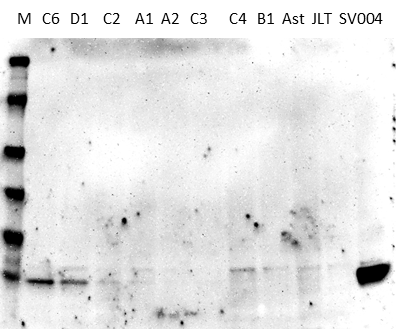

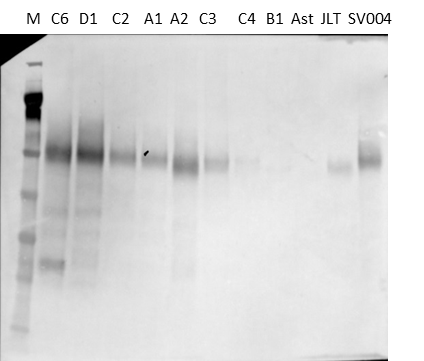


97 kDa

64 kDa

51 kDa

39 kDa

28 kDa

28 kDa

39 kDa

97 kDa

51 kDa

64 kDa

B

A
