# Supplementary material for: A versatile 2A peptide-based bicistronic protein expressing platform for the industrial cellulase producing fungus, Trichoderma reesei
Source: Biotechnol Biofuels. 2017 Feb 6;10:34. doi: 10.1186/s13068-017-0710-7 (PMC5294774; doi:10.1186/s13068-017-0710-7)
Supplement: Supplementary file 4 — Additional file 4. PCR analysis to determine the presence of cel7A, eGFP and both in the C2G transformants. Lanes M. Molecular weight marker (GeneRuler 1kb DNA ladder); A1, C2, C4, C5,D1, D3, A2 and A4, C2G transformant colonies; AST1114, Cel7A deleted T. reesei QM6A strain); JLT102A, AST1116 expressing native Cel7A under the eno promoter; SV001 and SV002, AST1116 expressing eGFP-2A-Cel7A; SV004, AST1116 expressing Cel7A-2A-eGFP. [file 13068_2017_710_MOESM4_ESM.docx]

Additional file 4.

PCR analysis to determine the presence of *cel7A*, *eGFP* and both in the C2G transformants. Lanes M. Molecular weight marker (GeneRuler 1kb DNA ladder); A1, C2, C4, C5,D1, D3, A2 and A4, C2G transformant colonies; AST1114, Cel7A deleted *T. reesei* QM6A strain); JLT102A, AST1116 expressing native Cel7A under the *eno* promoter; SV001 and SV002, AST1116 expressing eGFP-2A-Cel7A; SV004, AST1116 expressing Cel7A-2A-eGFP.


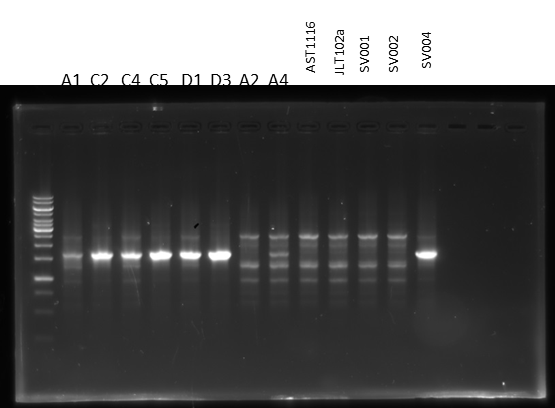
PCR1: Primers used Mf-PacI-F and FMDV-R1

Expected product size: 1659 bp

1.0 kb

1.5 kb

2.0 kb


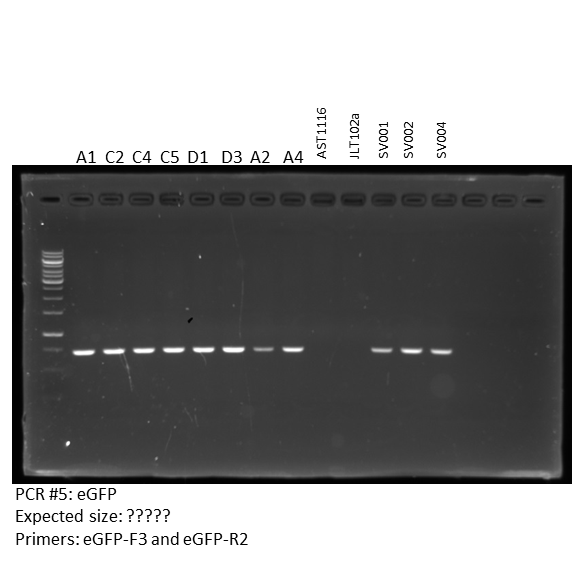


PCR2: Primers used eGFP-F3 and eGFP-R2

Expected product size: 706 bp

750 bp

500 bp


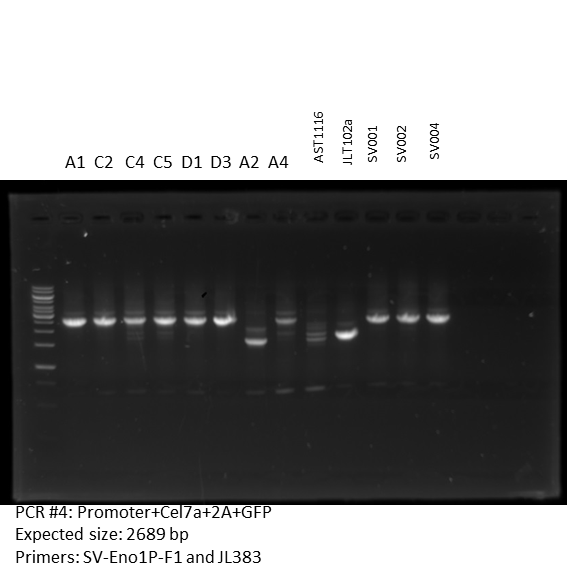


PCR3: Primers used Eno1P-F1 and JL383

Expected product size: 2689 bp

3.0 kb
