# Supplementary material for: A versatile 2A peptide-based bicistronic protein expressing platform for the industrial cellulase producing fungus, Trichoderma reesei
Source: Biotechnol Biofuels. 2017 Feb 6;10:34. doi: 10.1186/s13068-017-0710-7 (PMC5294774; doi:10.1186/s13068-017-0710-7)
Supplement: Supplementary file 5 — Additional file 5. PCR analysis to determine the presence of cel7A, eGFP and both in the G2C transformants. PCR analysis to determine the presence of cel7A, eGFP and both in the G2C transformants. Lanes M. Molecular weight marker (GeneRuler 1kb DNA ladder); C6, D1, C2, C3, A1, A2 C4 and B1, G2C transformant colonies; AST1114, Cel7A deleted T. reesei QM6A strain); JLT102A, AST1116 expressing native Cel7A under the eno promoter; SV001 and SV002, AST1116 expressing eGFP-2A-Cel7A; SV004, AST1116 expressing Cel7A-2A-eGFP. [file 13068_2017_710_MOESM5_ESM.docx]

Additional file 5.


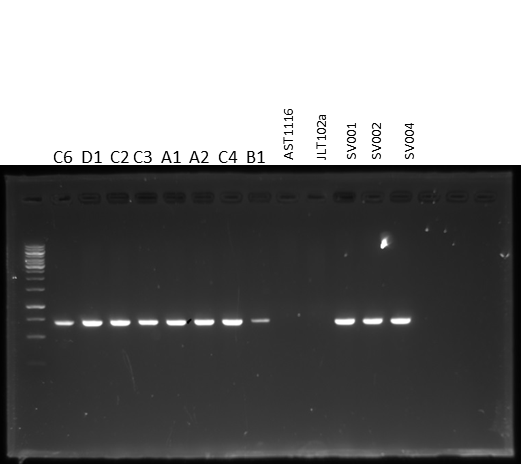
PCR analysis to determine the presence of *cel7A*, *eGFP* and both in the G2C transformants. PCR analysis to determine the presence of *cel7A*, *eGFP* and both in the G2C transformants. Lanes M. Molecular weight marker (GeneRuler 1kb DNA ladder); C6, D1, C2, C3, A1, A2 C4 and B1, G2C transformant colonies; AST1114, Cel7A deleted *T. reesei* QM6A strain); JLT102A, AST1116 expressing native Cel7A under the *eno* promoter; SV001 and SV002, AST1116 expressing eGFP-2A-Cel7A; SV004, AST1116 expressing Cel7A-2A-eGFP.

PCR1: Primers used eGFP-F3 and eGFP-R2

Expected product size: 706 bp

500 bp

750 bp


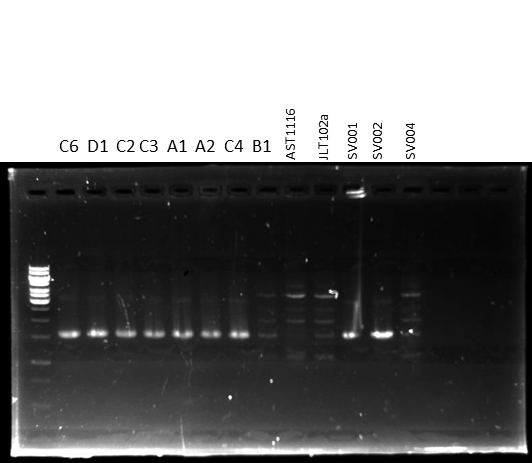


PCR2: Primers used cel7A-BamHI-F and JL387

Expected product size: 1601 bp

1 kb

1.5 kb

2.0 kb


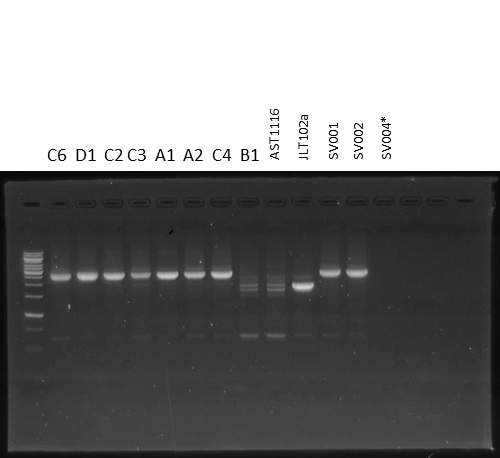
PCR3: Primers used Eno1P-F1 and JL383

Expected product size: 2689 bp

3.0 kb

* Although SV004 did not work in this PCR, the same PCR reaction worked in Additional file 4.
